# The interaction between E3 ubiquitin ligase Parkin and mitophagy receptor PHB2 links inner mitochondrial membrane ubiquitination to efficient mitophagy

**DOI:** 10.1016/j.jbc.2022.102704

**Published:** 2022-11-12

**Authors:** Shan Sun, Hongyu Hou, Guoqiang Ma, Qilian Ma, Ningning Li, Li Zhang, Chunsheng Dong, Mian Cao, Kin Yip Tam, Zheng Ying, Hongfeng Wang

**Affiliations:** 1 Jiangsu Key Laboratory of Neuropsychiatric Diseases and College of Pharmaceutical Sciences, Soochow University, Suzhou, Jiangsu, China.; 2 Faculty of Health Sciences, University of Macau, Taipa, Macau, China; 3 Key Laboratory of Nuclear Medicine, Ministry of Health, Jiangsu Key Laboratory of Molecular Nuclear Medicine, Jiangsu Institute of Nuclear Medicine, Wuxi, Jiangsu, China; 4 Insititutes of Biology and Medical Science, Soochow University, Suzhou, Jiangsu, China; 5 Programme in Neuroscience and Behavioural Disorders, Duke-NUS Medical School, Singapore, Singapore

**Keywords:** mitophagy, Parkin, PHB2, ubiquitination, MAP1LC3B/LC3B, (A/O), Antimycin A and Oligomycin A, EGFP, enhanced green fluorescent protein, GST, glutathione-s-transferase, IMM, inner mitochondrial membrane, MEFs cell, murine embryonic fibroblasts cell, MAP1LC3B/LC3B, microtubule associated protein 1 light chain 3 beta, OMM, outer mitochondrial membrane, OPTN, optineurin, PHB2, prohibitin 2, PLA, proximity ligation assay, TOM20, translocase of outer mitochondrial membrane 20, Ub, E3 ubiquitin, WT, wildtype

## Abstract

The autophagic clearance of mitochondria has been defined as mitophagy, which is triggered by mitochondrial damage and serves as a major pathway for mitochondrial homeostasis and cellular quality control. PINK1 and Parkin-mediated mitophagy is the most extensively studied form of mitophagy, which has been linked to the pathogenesis of neurodegenerative disorders, including Alzheimer’s disease, Parkinson’s disease, and amyotrophic lateral sclerosis. The current paradigm of this particular mitophagy pathway is that the ubiquitination of the outer mitochondrial membrane is the key step to enable the recognition of damaged mitochondria by the core autophagic component autophagosome. However, whether the inner mitochondrial membrane (IMM) is ubiquitinated by Parkin and its contribution to sufficient mitophagy remain unclear. Here, using molecular, cellular, and biochemical approaches, we report that prohibitin 2 (PHB2), an essential IMM receptor for mitophagy, is ubiquitinated by Parkin and thereby gains higher affinity to the autophagosome during mitophagy. Our findings suggest that Parkin directly binds to PHB2 through its RING1 domain and promotes K11- and K33-linked ubiquitination on K142/K200 sites of PHB2, thereby enhancing the interaction between PHB2 and MAP1LC3B/LC3B. Interestingly and importantly, our study allows us to propose a novel model in which IMM protein PHB2 serves as both a receptor and a ubiquitin-mediated base for autophagosome recruitment to ensure efficient mitophagy.

Autophagy mediates the degradation of dysfunctional and unwanted cellular components by lysosomes and plays an essential role in cell homeostasis (1). The selective autophagy of mitochondria, named mitophagy which is an important pathway for cleaning damaged mitochondria, has been identified that tightly linked to Alzheimer’s disease, Parkinson’s disease (PD), and amyotrophic lateral sclerosis (2). PINK1 is a serine/threonine kinase encoded by *PARK6* gene, and Parkin is an E3 ubiquitin (Ub) ligase encoded by *PARK2* gene (3). These two PD-associated proteins play critical roles in mitophagy. Structurally, Parkin consists of Ubl, RING0, RING1, IBR, REP, and RING2 domains (4). In healthy conditions for mitochondria, PINK1 is cleaved by PARL and then is released to the cytosol for proteasomal degradation. Meanwhile, Parkin is autoinhibited, and the E2-binding site on Parkin is repressed by Ubl and REP domains under normal condition. However, PINK1 proteolysis will be inhibited upon mitochondrial damage, triggering the accumulation of uncleaved full-length PINK1 on damaged mitochondria (5, 6). Accumulated PINK1 recruits and activates nearby Ub by phosphorylating it (7, 8, 9). After the interaction of phosphorylated Ub (pUb) and Parkin, the Ubl domain in Parkin can be released and phosphorylated by PINK1, resulting in the exposure of E2-binding site on Parkin (4, 10). Through the above series of conformational changes, Parkin retains on mitochondrial surface for transporting Ub to the outer mitochondrial membrane (OMM) proteins. In PINK1/Parkin-mediated mitophagy process, Parkin mainly assembles K6-, K11-, K48-, and K63-linked polyubiquitination chains (among all eight forms of Ub chains, including Met1 and seven lysine-linked chains) on OMM (3, 11). However, little is known about the ubiquitination of the inner mitochondrial membrane (IMM) and mitochondrial matrix proteins in PINK1/Parkin-mediated mitophagy.

During mitophagy, the autophagy receptors bind to ATG8 family proteins, including MAP1LC3/LC3 and GABARAP subfamilies in mammals, through LC3-interacting region (LIR) motifs (12, 13). With the help of ATG8 family proteins, the selective autophagy receptors, including OPTN and NDP52, link the polyubiquitinated OMM to autophagosomal membrane (12, 13, 14). Interestingly and importantly, previous study reported that prohibitin 2 (PHB2), an essential IMM protein that plays important roles in lifespan, neurodegenerative diseases, and cancer, directly interacts with MAP1LC3B/LC3B (hereinafter referred to as LC3) and functions as an IMM mitophagy receptor for efficient clearance of IMM and mitochondrial matrix after ubiquitination-driven OMM rupture (15). However, whether the autophagic engulfment of IMM is also regulated by Parkin-mediated ubiquitination remains unknown.

We therefore aimed to further explore the relationship between PHB2-associated mitophagy and Parkin-mediated ubiquitination. Now we report that Parkin can directly bind to IMM protein PHB2 through its RING1 domain and ubiquitinates the K142/K200 sites of PHB2 through K11- and K33-linked polyubiquitination. Moreover, ubiquitinated PHB2 interacts with LC3 more easily and accelerates the autophagic clearance of damaged mitochondria. Therefore, our data have significant implication for exploring the molecular mechanism of IMM protein ubiquitination in PINK1/Parkin-mediated mitophagy.

## Results

### PHB2 directly interacts with Parkin

**Figure 1:**
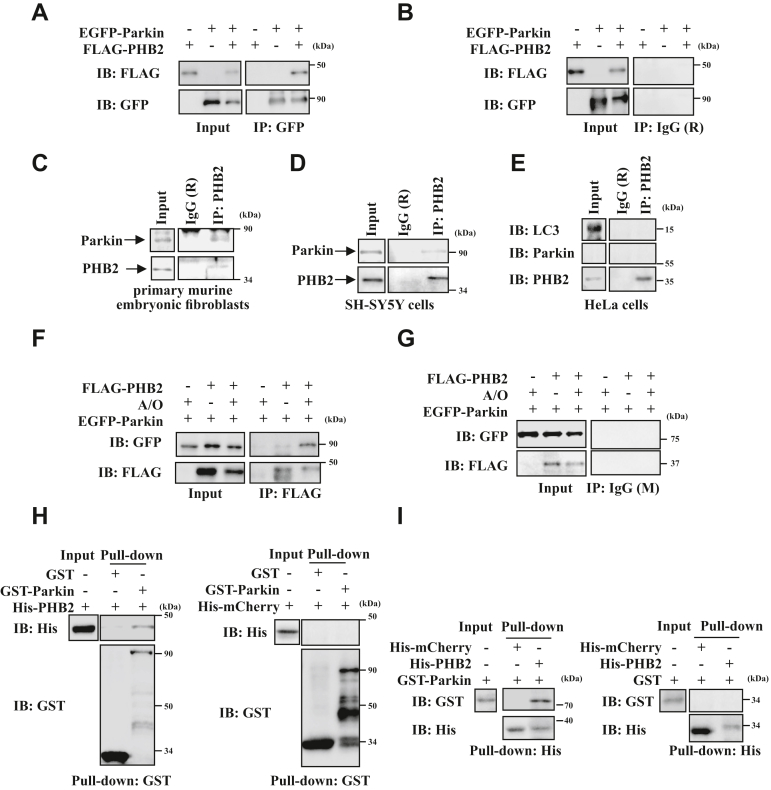
**Parkin directly interacts with PHB2, and the interaction is enhanced during mitophagy.***A* and *B*, HEK293T cells expressing EGFP vector or EGFP-Parkin with FLAG vector or FLAG-PHB2 were treated with 5 μg/ml A/O (Antimycin A and Oligomycin A) for 4 h. Cell lysates were subjected to immunoprecipitation analysis using anti-GFP antibody (*A*) or anti-IgG (R) antibody (*B*). Co-immunoprecipitated FLAG-PHB2 was detected using anti-FLAG antibody, n = 3. *C*–*E*, primary murine embryonic fibroblasts cells (*C*), SH-SY5Y cells (infected with lentivirus carrying mCherry-Parkin) (*D*) or HeLa cells (*E*) were treated with 5 μg/ml A/O for 4 h, and then cell lysates were subjected to immunoprecipitation analysis using anti-PHB2 antibody. Co-immunoprecipitated proteins were detected using anti-Parkin and anti-LC3 antibodies, n = 3. *F–G*, HEK293T cells expressing FLAG vector or FLAG-PHB2 with EGFP-Parkin were treated with DMSO or 5 μg/ml A/O for 4 h. Cell lysates were subjected to immunoprecipitation analysis using anti-FLAG antibody (*F*) or anti-IgG (M) antibody (*G*), and co-immunoprecipitated EGFP-Parkin was detected with anti-GFP antibody, n = 3. *H–I*, after purification by GST (*H*) or His (*I*) binding resin, GST-tagged (*H*) or His-tagged (*I*) proteins were separately mixed with His-PHB2 (*H*, *left region*), His-mCherry (*H*, *right region*), GST-Parkin (*I*, *left region*), or GST (*I*, *right region*). 10% input was loaded as control. The bound proteins were analyzed by immunoblot analysis with anti-GST or anti-His antibodies, n = 3. EGFP, enhanced green fluorescent protein; PHB2, prohibitin 2.

Previous studies demonstrated that PHB2 is an essential IMM receptor and plays an important role in the regulation of PINK1/Parkin-mediated mitophagy (15). We wondered whether PHB2 can be regulated by Parkin, the key player in mitophagy. To this end, we performed immunoprecipitation assay in HEK293T cells and found that PHB2 interacted with Parkin (Fig. 1, *A* and *B*) in response to Antimycin A and Oligomycin A (A/O) which is commonly used to induce mitophagy in cultured cells. Moreover, the association between Parkin and endogenous PHB2 was observed in response to A/O in murine embryonic fibroblasts (MEFs) (Fig. 1*C*) and SH-SY5Y cells (Fig. 1*D*), but not in HeLa cells (Fig. 1*E*), confirming that PHB2 indeed interacts with Parkin during mitophagy. To explore whether PHB2 can interact with Parkin under normal condition, we performed immunoprecipitation assay in HEK293T cells and found that PHB2 could weakly interact with Parkin. Moreover, Parkin-PHB2 interaction was strikingly increased in A/O-treated cells (Fig. 1, *F* and *G*). To further determine whether there is a direct interaction between PHB2 and Parkin, we performed *in vitro* GST (Fig. 1*H*) and His (Fig. 1*I*) pull-down assays, and the results showed that Parkin could directly interact with PHB2 *in vitro* (Fig. 1, *H* and *I*, left region), whereas His-mCherry or GST tag could not (Fig. 1, *H* and *I*, right region). Taken together, our results indicated that PHB2 directly interacts with Parkin *in vitro* and in cells undergo mitophagy.

### RING1 domain is required for Parkin to interact with PHB2

**Figure 2:**
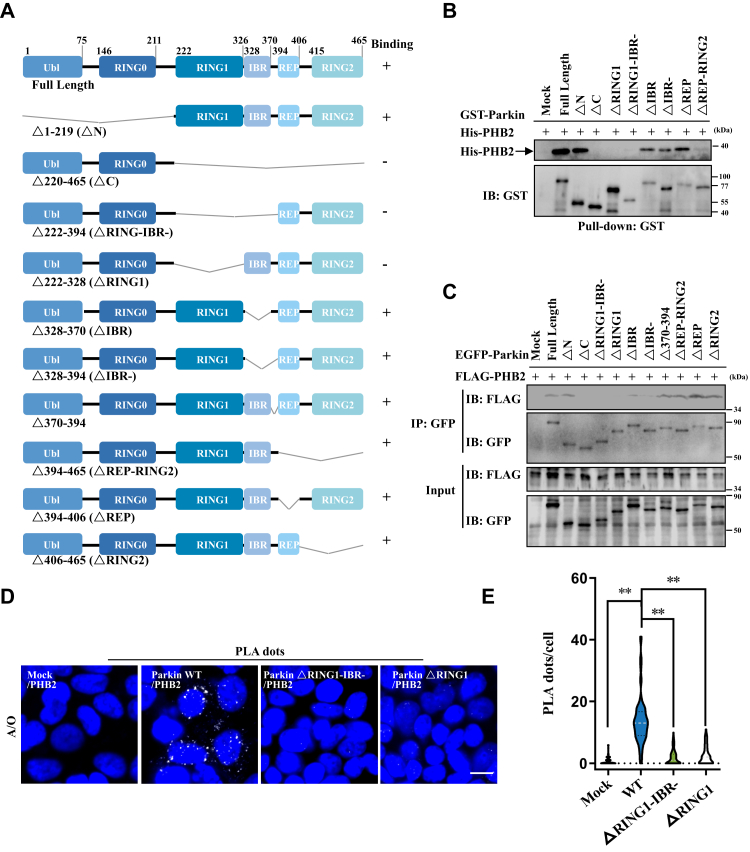
**RING1 domain is essential for Parkin–PHB2 interaction.***A*, schematic diagram of Parkin constructs used in this study. *B*, after purification by GST binding resin, different constructs of GST-Parkin (as indicated) were separately mixed with His-PHB2. Bound proteins were analyzed by immunoblot analysis with anti-GST and anti-PHB2 antibodies, n = 3. *C*, different constructs of EGFP-Parkin and FLAG-PHB2 were transfected into HEK293T cells, and then they were treated with 5 μg/ml A/O for 4 h. Cell lysates were processed for immunoprecipitation analysis using anti-GFP antibody and then were analyzed by immunoblot with indicated antibodies, n = 3. *D*, HEK293 cells expressing EGFP vector or EGFP-Parkin (WT, ΔRING1-IBR- or ΔRING1 mutant) were treated with 5 μg/ml A/O for 4 h, and then, the cells were fixed and stained with anti-GFP and anti-PHB2 antibodies in PLA experiment. The cells were visualized using Nikon microscope. Blue: nuclei (DAPI); white dots: PLA positive puncta. Scale bar, 10 μm. *E*, Violin plot of PLA puncta/cell in (*D*), n = 84, ∗∗*p* < 0.01, *One-way ANOVA* followed by post hoc Tukey’s tests. The gray dotted line represents the median, and the *black dotted line* represents the quartile. EGFP, enhanced green fluorescent protein; PLA, proximity ligation assay; PHB2, prohibitin 2.

To further determine which domain of Parkin is necessary for the interaction with PHB2, we generated truncated forms of Parkin containing different regions (Fig. S1*A*) and tested their interactions with PHB2. Immunoprecipitation in HEK293T cells and *in vitro* GST pull-down (Fig. S1, *B* and *C*) showed that the full-length Parkin and “RING1-IBR-” region could interact with PHB2. Therefore, we further generated a series of truncated mutants of Parkin lacking different regions (Fig. 2*A*) and analyzed their interactions with PHB2 using *in vitro* GST pull-down and immunoprecipitation assays in HEK293T cells and found that Parkin mutants that lacked RING1 domain could not interact with PHB2 (Fig. 2, *B* and *C*). We next used a proximity ligation assay (PLA) in A/O-treated HEK293 cells, and the results showed a dramatic decrease in PLA positive dots (reflected PHB2-Parkin interaction) when “RING1-IBR-” or “RING1” was depleted from Parkin (Figs. 2, *D*, *E* and S1*D*), indicating that Parkin binds to PHB2 through its RING1 domain.

### Parkin ubiquitinates the K142/K200 sites of PHB2 and produces K11 and K33 polyubiquitination linkages

**Figure 3:**
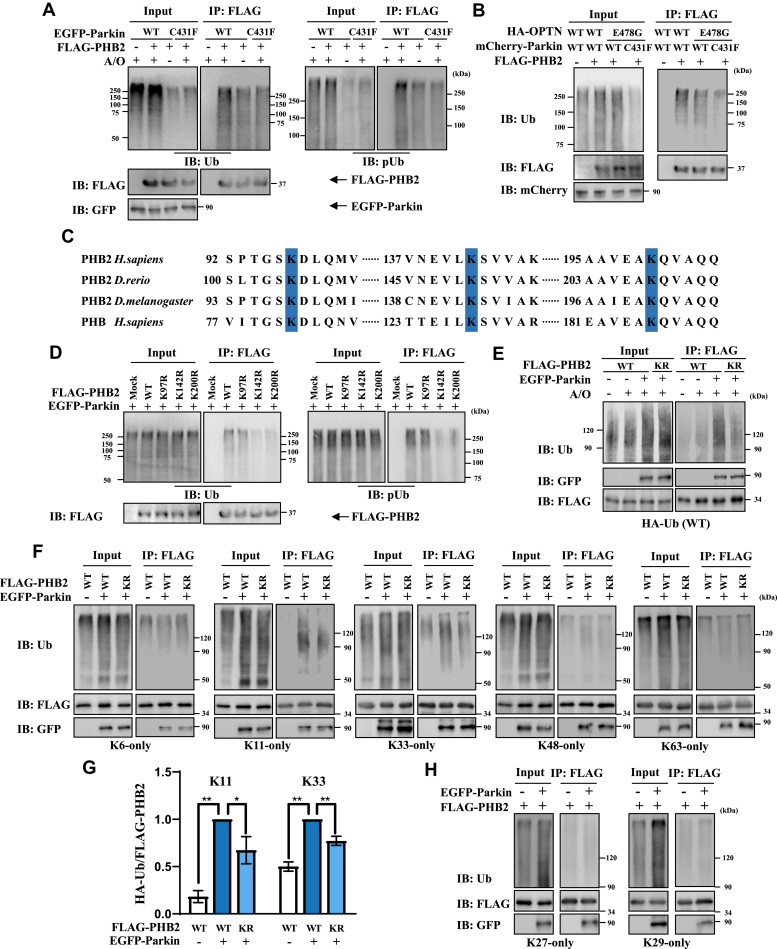
**Parkin promotes K11- and K33-linked ubiquitination of PHB2 on 142/200 sites.***A*, FLAG vector or FLAG-PHB2 with EGFP-Parkin (WT or C431F mutant) were transfected into HEK293T cells, followed by treatment with DMSO or 5 μg/ml A/O for 4 h. Cell lysates were subjected to immunoprecipitation analysis using anti-FLAG antibody and then were analyzed by immunoblot with indicated antibodies, n = 3. *B*, FLAG vector or FLAG-PHB2, HA-OPTN (WT or E478G mutant), along with mCherry-Parkin (WT or C431F mutant) were transfected into si-OPTN HEK293T cells, followed by treatment with 5 μg/ml A/O for 4 h. Cell lysates were subjected to immunoprecipitation analysis using anti-FLAG antibody and then were analyzed by immunoblot with indicated antibodies, n = 3. *C*, sequence alignment of PHB2 and PHB homologs across different species*. Blue shaded area* indicates the lysine residues which are evolutionarily conserved. *D*, FLAG vector or FLAG-PHB2 (WT, K97R, K142R or K200R mutant), along with EGFP-Parkin were transfected into HEK293T cells respectively, and then the cells were treated 5 μg/ml A/O for 4 h. Cell lysates were subjected to immunoprecipitation analysis using anti-FLAG antibody and then were analyzed by immunoblot with indicated antibodies, n = 3. *E*, EGFP vector or EGFP-Parkin, FLAG-PHB2 (WT or KR mutant) along with WT HA-Ub were transfected into HEK293T cells respectively, and then, the cells were treated with DMSO or 5 μg/ml A/O for 4 h. Cell lysates were subjected to immunoprecipitation analysis using anti-FLAG antibody and then were analyzed by immunoblot with indicated antibodies, n = 3. Here and hereafter KR mutant FLAG-PHB2 indicates K142R/K200R mutant FLAG-PHB2. *F*, EGFP vector or EGFP-Parkin, FLAG-PHB2 (WT or KR mutant) along with HA-Ub (K6, K11, K33, K48 or K63-only) were transfected into HEK293T cells respectively, followed by treatment with 5 μg/ml A/O for 4 h. Cell lysates were subjected to immunoprecipitation analysis using anti-FLAG antibody and then were analyzed by immunoblot with indicated antibodies, n = 3. *G*, quantification of co-immunoprecipitated Ub of K11 and K33 in (F), and the WT FLAG-PHB2 with GFP-Parkin expressing group were normalized as 100%. Data from three independent experiments were indicated as Means ± SD, ∗∗*p* < 0.01; ∗*p* < 0.05, *One-way ANOVA* followed by post hoc Tukey’s tests. *H*, the similar experiments (without KR mutant FLAG-PHB2) were separately performed in HA-Ub (K27 or K29-only) as (*F*), n = 3. EGFP, enhanced green fluorescent protein; OPTN, optineurin; PHB2, prohibitin 2.

As Parkin is an E3 Ub ligase and it interacts with PHB2, we wondered whether Parkin can ubiquitinate PHB2. We found that the ubiquitination of PHB2 was strikingly increased in A/O-treated HEK293T cells, but not in no-treatment or C431F mutant Parkin (a loss of function mutant)-expressing cells, suggesting that Parkin-mediated PHB2 ubiquitination requires full E3 activity of Parkin (Figs. 3*A* and S2*A*). OPTN is an important mitophagy receptor that binds to LC3 *via* LIR domain ([aa]169 to 230) and Ub *via* ubiquitin binding in ABIN and NEMO (UBAN) domain ([aa] 454 to 514). We further expressed E478G mutant OPTN (UBAN mutant that fails to translocate onto OMM during mitophagy) to exclude the additional LC3 recruitment and found that wildtype (WT) Parkin (along with WT or E478G mutant OPTN), but not C431F mutant Parkin, could ubiquitinate PHB2, suggesting that Parkin can ubiquitinate PHB2 in an E3 ligase activity-dependent manner, which is independent of OPTN (Figs. 3*B* and S2*B*).

To identify the Parkin-mediated ubiquitination sites on PHB2, we first performed sequence alignment analysis and found three evolutionarily conserved lysine residues (Fig. 3*C*). Next, we performed site-directed mutagenesis and immunoprecipitation assays in HEK293T cells and found mutation of K142 or K200 residues, but not K97, reduced ubiquitination of PHB2 (Figs. 3*D* and S2*C*). Our data suggested that Parkin promotes ubiquitination of PHB2 on both 142 and 200 lysines; therefore, we generated K142R/K200R double mutations (hereafter referred to as KR mutant PHB2) in PHB2 to have a better study. As shown in Fig. 3*E*, KR mutant PHB2 displayed reduced ubiquitination during mitophagy (Figs. 3*E* and S2*D*), suggesting that Parkin ubiquitinates PHB2 on the K142/K200 sites. Previous studies showed that Parkin ubiquitinates OMM protein through K6-, K11-, K48-, and K63-linked polyubiquitination chains to facilitate mitophagy (11). To further verify the type of Ub chains assembled to PHB2 in Parkin-expressing cells, we expressed different linkage types of Ub constructs (including WT and seven types of “K-only” mutant Ub constructs) and examined the ubiquitination level of WT and KR mutant PHB2. Our data showed Parkin-mediated K11- and K33-linked ubiquitination were downregulated in KR mutant PHB2-expressing cells, suggesting that Parkin promotes K11- and K33-linked ubiquitination of PHB2 on K142/K200 sites (Figs. 3, *F*–*H*, S2, *E* and *F*).

### Parkin ubiquitinates PHB2 and enhances the interaction between mitochondrial PHB2 and autophagosomal LC3

**Figure 4:**
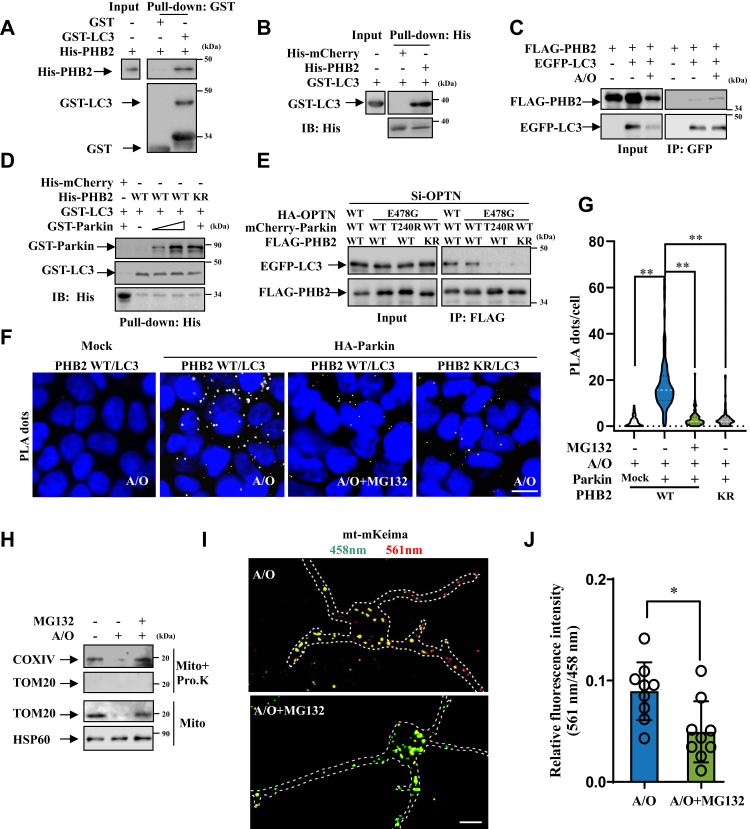
**Parkin-mediated ubiquitination enhances the association of PHB2 with LC3 upon proteasomal-driven OMM rupture.***A* and *B*, after purification by GST or His binding resin, purified GST-tagged (*A*) or His-tagged (*B*) proteins were mixed with His-PHB2 (*A*) or GST-LC3 (*B*). 10% input was loaded as control, and the bound proteins were analyzed by immunoblot analysis using anti-His and anti-GST antibodies, n = 3. *C*, HEK293T cells expressing EGFP vector or EGFP-LC3 with FLAG-PHB2 were treated with DMSO or 5 μg/ml A/O for 4 h. Cell lysates were subjected to immunoprecipitation analysis using anti-GFP antibody and then were analyzed by immunoblot with anti-FLAG and anti-GFP antibodies, n = 3. *D*, after purification by His binding resin, purified His-tagged proteins were mixed with GST-LC3, with or without different concentration of GST-Parkin. The bound proteins were analyzed by immunoblot analysis with anti-LC3, anti-Parkin and anti-His antibodies, n = 3. *E*, WT or E478G mutant HA-OPTN, WT or T240R mutant mCherry-Parkin, WT or KR mutant FLAG-PHB2 with EGFP-LC3 were transfected into si-OPTN HEK293T cells, followed by treatment with 5 μg/ml A/O for 4 h. Cell lysates were subjected to immunoprecipitation analysis using anti-FLAG antibody and then were analyzed by immunoblot with anti-FLAG and anti-GFP antibodies, n = 3. *F*, FLAG-PHB2 (WT or KR mutant) with or without HA-Parkin were transfected into HEK293 cells, followed by treatment with 5 μg/ml A/O for 4 h with or without MG132 (10 μM, pretreated for 8 h). Then the cells were fixed and stained with anti-FLAG and anti-LC3 antibodies in PLA experiment. The cells were visualized using Nikon microscope. *Blue*: nuclei (DAPI); *white dots:* PLA puncta. Scale bar, 10 μm. *G*, Violin plot of PLA puncta/cell in (*F*), n = 84, ∗∗*p* < 0.01, *One-way ANOVA* followed by post hoc Tukey’s tests. The *gray dotted line* represents the median, and the *black dotted line* represents the quartile. *H*, after transfected with mCherry-Parkin and treated with 5 μg/ml A/O for 4 h with or without the combination of MG132 (10 μM, pretreated for 8 h), HEK293T cells were collected for mitochondria extraction. Then the mitochondria were treated with or without 10 μg/ml proteinase K for 30 min on ice before subjected to immunoblot analysis using anti-COXIV, anti-TOM20, and anti-HSP60 antibodies, n = 3. *I*, primary cortical neurons were transfected with lentiviral mt-mKeima and treated with 5 μg/ml A/O for 2 h with or without MG132 (10 μM, pretreated for 10 h). Then, the cells were visualized using confocal microscopy. Scale bar, 10 μm. *J*, quantification of 561 nm *versus* 458 nm channel signal in figure (*I*). Data were collected from nine cells from three independent experiments and were represented as means ± S.D., ns, not significantly different; ∗*p* < 0.05, t test. A/O, Antimycin A and Oligomycin A; EGFP, enhanced green fluorescent protein; OPTN: optineurin; PLA, proximity ligation assay; PHB2, prohibitin 2; TOM20, translocase of outer mitochondrial membrane 20.

As previous study reported that PHB2 interacts with LC3 after OMM rupture (15), we wondered whether the ubiquitination of PHB2 can affect the interaction between PHB2 and LC3. First, we confirmed that PHB2 could interact with LC3 *in vitro* (Fig. 4, *A* and *B*) and in HEK293T cells (Figs. 4*C* and S3*A*), but not in Parkin-deficient HeLa cells (Fig. 1*E*). Next, we examined whether Parkin alone can affect PHB2-LC3 interaction without Ub and found that the interaction between PHB2 and LC3 was not affected by co-incubation with different amounts of Parkin *in vitro*, suggesting that Parkin and LC3 does not competitively bind to PHB2, and Parkin cannot enhance PHB2-LC3 interaction through direct association with PHB2 (Fig. 4*D*). We also found that KR mutant PHB2 did not directly affect PHB2-LC3 interaction *in vitro* (without Ub), further supporting the conclusion that Parkin-mediated ubiquitination on K142/K200 sites affects PHB2-LC3 interaction (Fig. 4*D*).

Previously studies showed that OPTN not only binds to LC3 *via* LIR but also binds to Ub *via* UBAN during mitophagy (12, 13). To exclude the additional LC3 recruitment by OPTN (OMM mitophagy receptor), we performed immunoprecipitation using si-OPTN (knockdown of *OPTN*) HEK293T cells expressing WT or E478G mutant OPTN and found that PHB2-LC3 interaction was not affected by E478G mutation in OPTN but was decreased when expressing T240R mutant Parkin (loss of function mutant) or KR mutant PHB2 (Figs. 4*E* and S3*B*). Moreover, PLA assays also showed that PHB2-LC3 interaction was decreased in HEK293 cells expressing KR mutant PHB2 or without exogenous Parkin expression, indicating that ubiquitination of PHB2 enhances PHB2-LC3 interaction independently of OPTN (Figs. 4, *F*, *G* and S3*C*).

Given that LC3 can translocate onto IMM after OMM rupture in mitophagy (15), we next explored the relationship between proteasome-mediated OMM rupture and Parkin-PHB2-mediated mitophagy. PLA assay indicated that the interaction of LC3 and PHB2 was lower upon proteasomal inhibition (MG132 treatment) in HEK293 cells, suggesting the recruitment of LC3 onto IMM depends on proteasome-mediated OMM rupture (Fig. 4, *F* and *G*). Also, we found that proteinase K, an external protease which can digest IMM proteins when OMM is ruptured (5), could digest OMM protein translocase of outer mitochondrial membrane 20 (TOM20), but failed to digest IMM protein COXIV after proteasomal inhibition in A/O-treated HEK293T cells, suggesting that proteasomal degradation can lead to OMM rupture in mitophagy (Fig. 4*H*). In addition, mt-mKeima fluorescent reporter assay (16) was used to analyze mitophagic flux and showed that proteasomal inhibition significantly blocked mitophagic flux in cortical neurons (the 561 nm red dots of mt-mKeima reflects mitochondria in the acidic lysosomes, which indicate mitophagic flux) (Fig. 4, *I* and *J*). Taken together, these data suggested that proteasomal degradation is critical for OMM rupture and subsequent autophagic degradation of the rest of mitochondria.

**Figure 5:**
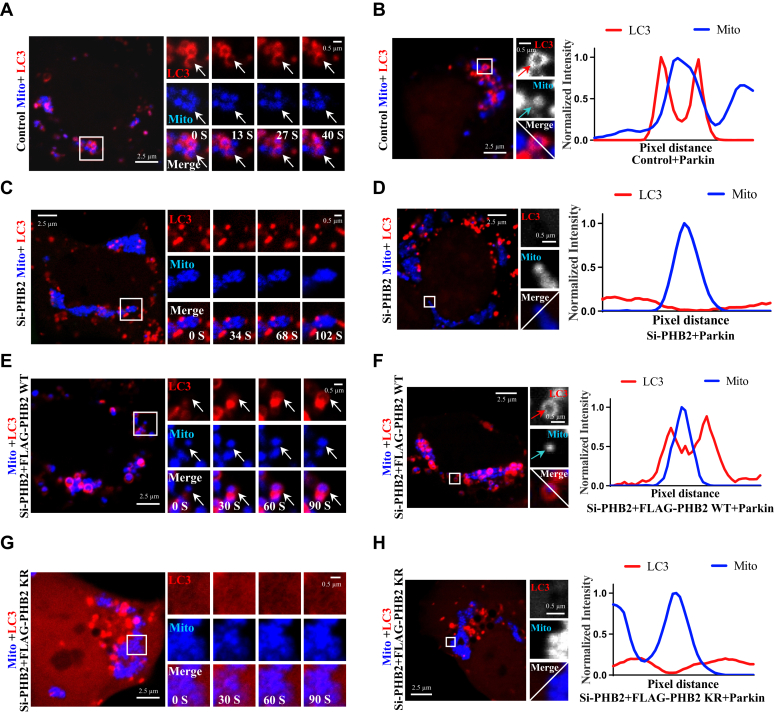
**Parkin-mediated ubiquitination of PHB2 is required for the recruitment of autophagosomes onto the mitochondria.***A*–*H*, HEK293 cells were transfected with control or si-PHB2 and then were re-transfected with FLAG-Parkin, BFP-Mito, EGFP-OPTN, and mCherry-LC3 with or without FLAG-PHB2 (WT or KR mutant). After treated with 5 μg/ml A/O for 6 h, the cells were analyzed by multichannel live cell imaging using confocal microscopy. Regions in white box were magnified on the right side. *White arrows* indicated that LC3 “rings” stably localized on the surface of damaged mitochondria at different times in (*A*) and (*E*). The fluorescence intensity of the paint line (*white line*) was measured in (*B*, *D*, *F*, and *H*). LC3 (*red arrow*) could surround the damaged mitochondrion (*blue arrow*) in (*B* and *F*), but not in (*D* and *H*). Scale bars, 2.5 μm. A/O, Antimycin A and Oligomycin A; EGFP, enhanced green fluorescent protein; OPTN: optineurin; PHB2, prohibitin 2; TOM20: translocase of outer mitochondrial membrane 20.

We further examined the mitochondrial recruitment of LC3 using microscopy imaging assay in HEK293 cellular model of mitophagy and found that LC3 could surround mitochondria in control (Fig. 5*A*) or si-PHB2/PHB2-WT (re-expression of WT PHB2 in si-PHB2 cells) (Fig. 5*E*) cells, but not in si-PHB2 cells (Fig. 5*C*), si-PHB2/PHB2-KR cells (re-expression of KR PHB2 in si-PHB2 cells) (Fig. 5*G*), or control cells without exogenous Parkin expression (Fig. S4*A*) cells. Also, we identified clear LC3 “rings” surrounding mitochondria “granules”, which represents robust recruitment of LC3 onto damaged mitochondria. Importantly, LC3 could be easily recruited onto damaged mitochondria in control (Fig. 5*B*) and si-PHB2/PHB2-WT (Fig. 5*F*) cells, but not in PHB2-deficient cells (Fig. 5*D*), si-PHB2/PHB2-KR cells (Fig. 5*H*), or without exogenous Parkin expressing (Fig. S4*B*) cells (Fig. S4, *C* and *D*). These data suggested that the recruitment of autophagosome to IMM is dependent on the Parkin-mediated ubiquitination of PHB2.

Next, we sought to analyze whether Parkin-mediated ubiquitination of PHB2 is associated with proteasomal degradation in HEK293T cells, and we found that autophagy inhibitor (bafilomycin A_1_) treatment could completely block A/O-induced PHB2 degradation (Fig. S5, *A* and *B*), suggesting that PHB2 is fully degraded by autophagy (lysosomes), but not proteasomes during mitophagy. Relevant to this, KR mutation also fully blocked PHB2 turnover (Fig. S5, *C* and *D*), suggesting that Parkin-mediated PHB2 ubiquitination mainly drives lysosomal but not proteasomal degradation of PHB2 (along with the mitochondria). Therefore, ubiquitinated PHB2 is mainly degraded by lysosomes but not proteasomes during Parkin-mediated mitophagy. Along with the other data, these results suggested that after proteasome-mediated OMM rupture, IMM protein PHB2 is exposed to the cytoplasm and ubiquitinated by Parkin for efficient association with LC3, which further triggers mitophagic clearance of the entire mitochondria.

### Parkin ubiquitinates the inner membrane receptor PHB2 to promote efficient mitophagy

**Figure 6:**
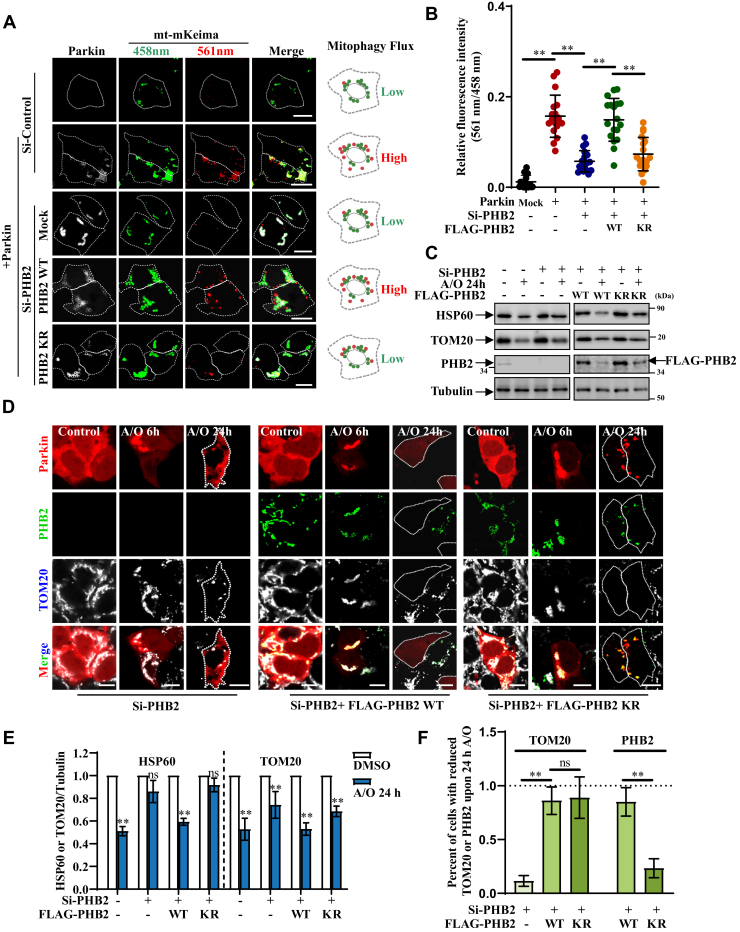
**Parkin-mediated ubiquitination of PHB2 ensures efficient mitophagy.***A*, HEK293 cells were transfected with control or si-PHB2 and then were retransfected with mt-mKeima, FLAG-PHB2 (mock, WT or KR mutant) with or without EGFP-Parkin. After treated with 5 μg/ml A/O for 4 h, the cells were analyzed by confocal microscopy, and the cartoon model on the right elucidated the relative levels of mitophagy flux. Scale bars, 10 μm. *B*, quantification of 561 nm *versus* 458 nm channel signal in figure (*A*). Data from 18 cells from three independent experiments were represented as means ± S.D., ∗∗*p* < 0.01. *One-way ANOVA* followed by post hoc Tukey’s tests. *C*, HEK293T cells were transfected with control or si-PHB2 and then were re-transfected with mCherry-Parkin and FLAG-PHB2 (mock, WT or KR mutant). Then the cells were treated with DMSO or 1 μg/ml A/O for 24 h, and cell lysates were analyzed by immunoblot analysis using anti-HSP60, anti-TOM20, anti-PHB2 (for detecting endogenous PHB2), anti-FLAG (for detecting exogenous PHB2), and anti-Tubulin antibodies. *D*, HEK293 cells with si-PHB2 were re-transfected with mCherry-Parkin and FLAG-PHB2 (mock, WT, or KR mutant), followed by treatment with DMSO, 5 μg/ml A/O for 6 h or 1 μg/ml A/O for 24 h. The cells were fixed and stained with anti-TOM20 and anti-FLAG antibodies and then were analyzed by confocal microscopy. The *dashed white line* in 24 h A/O treatment groups is the exogenous Parkin-expression cells outline. Scale bars, 10 μm. *E*, HSP60 and TOM20 protein levels in cells with 24 h A/O treatment in figure (*C*) were normalized to Tubulin, compared with nontreated group. Data were collected from three independent experiments and were represented as means ± S.D., ns, not significantly different; ∗∗*p* < 0.01, *t* test. *F*, quantification of the percent of cells with reduced TOM20 and PHB2 in figure (*D*). Data collected from three independent experiments were represented as means ± S.D., ns, not significantly different; ∗∗*p* < 0.01, *One-way ANOVA* followed by post hoc Tukey’s tests. A/O, Antimycin A and Oligomycin A; EGFP, enhanced green fluorescent protein; PHB2, prohibitin 2; TOM20, translocase of outer mitochondrial membrane 20.

To test the effect of PHB2 ubiquitination on mitophagic flux, we performed mt-mKeima experiment in HEK293 (Fig. 6, *A* and *B*) and SH-SY5Y (Fig. S6*A*) cells. Upon treatment with A/O, knockdown *PHB2* significantly reduced mitophagic signal induced by Parkin and impaired mitophagy could be rescued by re-expressing WT PHB2, but not KR mutant PHB2, suggesting that Parkin-mediated ubiquitination of PHB2 is essential for mitophagy. Quantification analysis also revealed that Parkin-mediated ubiquitination of PHB2 affected mitophagy over time (Figs. S4, *C–D* and S6*B*). To further analyze the clearance of impaired mitochondria, we performed long term A/O-treatment assay in HEK293T (Fig. 6, *C* and *E*) and HEK293 cells (Fig. 6, *D* and *F*). The results showed that most of damaged mitochondria could be removed in control and si-PHB2/PHB2-WT cells, but not in si-PHB2 or si-PHB2/PHB2-KR cells, indicating Parkin-mediated ubiquitination of PHB2 promotes robust clearance of damaged mitochondria. Relevant to this, our results showed that TOM20 was likely to be degraded by proteasomes (along with other OMM proteins) in si-PHB2/PHB2-WT and si-PHB2/PHB2-KR cells after long-term A/O treatment, further suggesting that Parkin-mediated ubiquitination of PHB2 does not affect proteasomal degradation of OMM proteins (Fig. 6, *C*–*F*). Meanwhile, IMM protein PHB2 itself (along with the entire mitochondria) was not cleared in si-PHB2/PHB2-KR cells and still co-localized with Parkin on the remaining mitochondria that form aggregate-like structures upon 24h A/O treatment (Fig. 6, *D* and *F*). It was worthy to note that a recent study interestingly showed that PHB2 can regulate PINK1 stabilization, thereby promoting PINK1/Parkin-mediated mitophagy (17). We therefore compared PINK1 protein level in si-PHB2/PHB2-WT with si-PHB2/PHB2-KR cells upon A/O-treatment. Our data showed that *PHB2* knockdown indeed reduced PINK1 level, whereas re-expression of PHB2-WT and PHB2-KR restored PINK1 level to a similar extent (Fig. S6*C*). Thus, our data indicated that the Parkin-mediated ubiquitination of PHB2 on K142/200 sites does not influence PINK1 stabilization but plays important role in mitochondrial recruitment of autophagosomes, suggesting that PHB2 has two separate functions in PINK1/Parkin-mediated mitophagy pathway.

## Discussion

Among all the genetic causes of PD, mutations in PINK1 and Parkin cause early-onset PD (3, 18). In addition, suppression of PINK1 and Parkin proteins leads to motor neuron and muscle degeneration in *Drosophila*, indicating that these two proteins can function in same pathway, and loss of their function will lead to neurodegeneration (19). Given that PINK1 and Parkin play essential roles in mitochondrial quality control, the autophagic clearance of IMM and mitochondrial matrix driven by PHB2 may participate in PD pathogenesis in association with PINK1/Parkin. However, little is known about the effect of PINK1/Parkin on IMM protein PHB2 during mitophagy. In the present study, we demonstrate that Parkin can bind and ubiquitinate PHB2 after OMM rupture by proteasomes, thereby promoting the efficient mitophagy by increasing the binding affinity of LC3 to PHB2.

Unlike previous studies focusing on the OMM, our findings propose a novel mechanism that Parkin-mediated ubiquitination also affects the IMM. Along with the recent interesting study which showed that PHB2 can stabilize PINK1 by regulating PARL and promote the accumulation of Parkin on mitochondria to enhance mitophagy (17), our findings not only strengthen the connection between PINK1/Parkin-mediated mitophagy and PHB2 (Figs. 1 and 2) but also highlight the contribution of PHB2 ubiquitination to PINK1/Parkin-mediated mitophagy (Figure 3, Figure 4, Figure 5, Figure 6), which is independent of PHB2-associated PINK1 stabilization. Therefore, PHB2 plays important roles in regulating mitochondrial quality control, and biochemical modification on PHB2 contributes to the efficient mitophagic clearance of the entire mitochondria. Furthermore, it has been reported that PINK1/Parkin-mediated mitophagic ubiquitination of OMM is a positive-feedback system to increase the recognition of OMM by autophagosome (11). Relevant to this, our finding of IMM-Ub system may also suggest a positive-feedback system for further facilitating the overall mitochondrial ubiquitination and enhance the covering of mitochondria by autophagosome.

**Figure 7:**
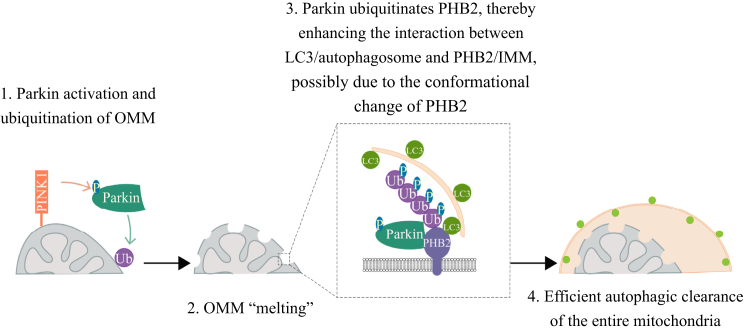
**Schematic model illustrating the contribution of Parkin-mediated IMM PHB2 ubiquitination to efficient mitophagy.** Step 1: upon mitochondrial damage, PINK1 accumulates on mitochondrion to phosphorylate ubiquitin and Parkin, and then OMM proteins are ubiquitinated by Parkin. Step 2: ubiquitinated OMM proteins are degraded by proteasomes, leading to OMM rupture (melting). Step 3: Parkin promotes ubiquitination of IMM protein PHB2 on K142/K200 sites, thereby promoting the association between PHB2 and LC3-labeled autophagosome, possibly due to the conformational change of PHB2. Step 4: the entire mitochondrion is degraded by autophagy-lysosome pathway. IMM, inner mitochondrial membrane; OMM, outer mitochondrial membrane; PHB2, prohibitin 2.

Interestingly, several important studies reported that OPTN is the key mitophagy receptor that binds to Atg8 proteins (such as LC3) *via* LIR domain and Ub *via* UBAN domains (12, 13). Through these interactions, OPTN can link LC3-labeled autophagosome to ubiquitinated mitochondria. Notably, Atg8 proteins can play an amplifier role in OMM recruitment of mitophagy receptors, since they are not only recruited by the receptors such as OPTN, but also draw more receptors on the OMM to boost the further cycles and facilitate the formation of OPTN-mediated mitophagosome (20). Therefore, PHB2 may help to recruit OPTN on the IMM *via* two ways: (1) PHB2-bound LC3 may pull OPTN on IMM in a LIR-dependent manner, similar to the OMM-LC3 amplifying machinery (20) and (2) Ub chains on PHB2 may recruit OPTN on the IMM in a UBAN-dependent manner. To dissect the precise molecular mechanism underlying PHB2 ubiquitination-mediated autophagosome (LC3) recruitment, we exclude the contribution of Ub-bound OPTN by expressing E478G mutant OPTN (UBAN mutant) which blocks the additional LC3 recruitment through inhibiting the potential OPTN-Ub association on PHB2. In addition, as indicated by the experiments using T240R mutant Parkin or KR mutant PHB2, we find that the ubiquitination of PHB2 can enhance the binding affinity of LC3 to PHB2 (Figs. 4 and 5), possibly due to the conformational change of PHB2 after ubiquitination (Fig. 7, step 3). Given the fact that multiple types of protein ubiquitination drive diverse effects on the substrate, including the change of protein degradation, localization, activity, and protein–protein interaction (21), our results shed the light to the function of IMM protein ubiquitination, suggesting a noncanonical role of Parkin-mediated ubiquitination in mitophagy pathway.

Notably, proteasome-initiated and lysosome-executed degradation are two essential steps for PINK1/Parkin-mediated mitophagy (15, 22, 23). When mitochondria are damaged, multiple OMM proteins, including mitochondrion fusion-associated proteins Mfn1 and Mfn2, are extensively ubiquitinated and degraded by Parkin-mediated proteasomal degradation to inhibit mitochondrial refusion, thereby isolating dysfunctional mitochondria and promoting efficient mitophagy (24). After the “melting” of OMM, IMM, and mitochondrial matrix are exposed and eventually degraded by autophagy-lysosome pathway (22). In conclusion, our results show that not only proteasome-dependent rupture of the OMM is important for mitophagy (Fig 4, *F–J*) but also the ubiquitination of IMM protein PHB2 is necessary for efficient clearance of mitochondria (Figs 5 and 6). Furthermore, as an IMM protein, PHB2 is mainly degraded by autophagy-lysosome pathway, but not by proteasomes (Fig. S5). These results suggest that PHB2 can be exposed to the cytosol and biochemically modified during mitophagy and plays a critical role as an IMM receptor in depletion of the entire mitochondria (Fig. 7).

Taken together, our study reveals that the Ub-signal on IMM is essential for the integrity of PINK1/Parkin-mediated autophagic clearance of mitochondria (Fig. 7). It is not only helpful for understanding the basic mechanism of mitophagy driven by Parkin but also identifies PHB2 as a potential player in neurodegenerative diseases that are associated with dysfunctional mitochondrial quality control, including PD and amyotrophic lateral sclerosis.

## Experimental procedures

### Plasmid constructs and siRNAs

Phage-CMV-C-HA-PHB2 was kindly provided by Dr Yingjie Xu (Shanghai Jiao Tong University). HA-Ub (WT, K6 only, K11 only, K27 only, K29 only, K33 only, K48 only, and K63 only) were generously provided by Dr Hui Zheng (Soochow University); enhanced green fluorescent protein (EGFP), mt-mKeima, mCherry-Parkin (WT, T240R and C431F), BFP-Mito, EGFP-Parkin (WT, ΔN and ΔC), GST-Parkin (WT, ΔN and ΔC), EGFP-OPTN, HA-OPTN (WT and E478G), 3 × FLAG-Parkin, and EGFP-LC3 plasmids were described previously (25, 26, 27, 28). mCherry-hLC3B-pcDNA3.1 was a gift from David Rubinsztein (Addgene plasmid #40827). His-PHB2 was created by subcloning PCR product with XhoI/BglⅡ sites from Phage-CMV-C-HA-PHB2 into pET-15b vector (Novagen, 69661–3) at XhoI/BamHI sites. GST-LC3 was created by subcloning PCR product with EcoRI/SalI sites into pGEX-5X-1 vector (Amersham Biosciences, 27–4584–01). 3 × FLAG-PHB2 was created by subcloning PCR product with EcoRI/BglⅡ sites from Phage-CMV-C-HA-PHB2 into p3 × FLAG-myc-CMV14 vector (Sigma, E6151) at EcoRI/BamHI sites. His-mCherry was created by subcloning PCR product with NdeI/XhoI sites from mCherry-Parkin into pET-15b vector (Novagen, 69661–3). HA-Parkin was created by subcloning PCR product with BamHI/EcoRI sites from EGFP-Parkin into pKH3. EGFP-Parkin (C431F) was created by subcloning from mCherry-Parkin (C431F) into pEGFP-C1 vector using EcoRI/SalI sites.

Double lysine mutants of p3 × FLAG-myc-CMV14-PHB2 (K97R/K142R, K97R/K200R, K142R/K200R) were generated by the site-directed mutagenesis using MutanBEST kit (Takara, R401) with following primers: 5′-GGAGCCTGTAGGGGAGGAGAT-3′ and 5′-AGAGACCTACAGATGGTGAAT-3′ for K97R mutant; 5′-CCTGAGCACCTCGTTGACAAT-3′ and 5′-AGTGTGGTGGCCAAGTTCAAT-3′ for K142R mutant; 5′-GGCCACTTGTCTGGCTTCTA-3′ and 5′-CAGCAGGAGGCCCAGCGG-3′ for K200R mutant. Mutants of EGFP-Parkin and GST-Parkin were generated by the site-directed mutagenesis using MutanBEST kit (Takara) with following primers: 5′-AAAGCTGTTGTAGATTGATCTA-3′ and 5′-TAGGTCGACGGTACCGCG-3′ for EGFP-Parkin (Ubl); 5′-AAAGCTGTTGTAGATTGATCTA-3′ and 5′-TAGGTCGACTCGAGCGGC-3′ for GST-Parkin (Ubl); 5′-CATGGGGATCCCACGACCT-3′ and 5′-TATGTGTATTGCAAAGGCCCC-3′ for GST-Parkin (RING0) from GST-Parkin (ΔC); 5′-GACTCTGTAGGCCTG-3′ and 5′-TAGGTCGACTCGAGCGGC-3′ for GST-Parkin (RING1+IBR) and EGFP-Parkin (RING1+IBR) from GST-Parkin (ΔN); 5-CATGGGGATCCCACGACCT-3′ and 5′-GAAAGAGCCGCCGAGCAG-3′ for GST-Parkin (REP + RING2) from GST-Parkin (ΔN); 5′-TTCCTTGTCAGAGGTGGG-3′ and 5′-GGCGTGTTATGCCCCCGC-3′ for EGFP-Parkin (ΔRING1) and GST-Parkin (ΔRING1); 5′-TTCCTTGTCAGAGGTGGG-3′ and 5′-GAAAGAGCCGCCGAGCAG-3′ for EGFP-Parkin (ΔRING1-IBR-) and GST-Parkin (ΔRING1-IBR-); 5′-CATCTGCAGGACACACTCC-3′ and 5′-GCGTACCATGAAGGG-3′ for EGFP-Parkin (ΔIBR) and GST-Parkin (ΔIBR); 5′-CATCTGCAGGACACACTCC-3′ and 5′-GAAAGAGCCGCCGAGCAG-3′ for EGFP-Parkin (ΔIBR-) and GST-Parkin (ΔIBR-); 5′-GACTCTGTAGGCCTG-3′ and 5′-TCCAAAGAAACCATC-3′ for EGFP-Parkin (ΔREP) and GST-Parkin (ΔREP); 5′-TTTACATTCCCGGCAGAA-3′ and 5′-GAAAGAGCCGCCGAGCAG-3′ for EGFP-Parkin (Δ370–394); 5′- TGCTTCCCAACGAGCCTG -3′ and 5′-TAGGTCGACGGTACCGCG-3′ for EGFP-Parkin (ΔRING2); 5′-GACTCTGTAGGCCTG-3′ and 5′-TAGGTCGACTCGAGCGGC-3′ for GST-Parkin (ΔREP-RING2); 5′-GACTCTGTAGGCCTG-3′ and 5′- TAGGTCGACGGTACCGCG-3′ for EGFP-Parkin (ΔREP-RING2). EGFP-Parkin (RING0) and EGFP-Parkin (REP + RING2) digested by restriction enzyme from GST-Parkin (RING0) and GST-Parkin (REP + RING2) with BamHI/SalI sites into pEGFP-C2 vector at BglⅡ/SalI sites. All the plasmids were sequenced by Synbio Technologies.

The following siRNAs (GenePharma) were used: Negative control: A nontargeting oligonucleotide; Human PHB2: 5′-GUGAUUUCCUACAGUGUUGUUCCCT-3′; Human OPTN: 5′-CUUCGAACAUGAGGAGUUATT-3′.

### Cell culture, transfection, and drug treatment

Human embryonic kidney 293 (HEK293) cells, human embryonic kidney 293T (HEK293T) cells, HeLa cells, and SH-SY5Y cells were cultured in DMEM medium (90%, Gibco, 11995500) containing fetal bovine serum (FBS) (10%, Gibco, 10099141C), streptomycin (100 μg/ml), and penicillin (100 U/ml) (Gibco, 15140122).

MEFs cells and primary cortical neurons culture was described previously (29). Briefly, MEFs and cortical neurons were respectively obtained from epithelium and cortex of C57BL/6 mouse embryos at embryonic day 17. The dissociated MEFs were cultured in medium with 90% DMEM and 10% FBS. The dissociated cortical neurons were cultured in neurobasal medium (Gibco, 21103049) with 10% FBS for 8 h. Then, the medium was replaced to supplemented neurobasal medium without serum.

SH-SY5Y cells were infected with lentivirus carrying mCherry-Parkin (HanBio Biotechnology). Lipofectamine 2000 reagent (Invitrogen, 11668019) wrapped plasmids were transfected into the cells maintained in Opti-MEM (Gibco, 31985070) and none serum medium. The following chemicals were used for treatment: Antimycin A (1 or 5 μg/ml, Sigma, A8674), oligomycin A (1 or 5 μg/ml, Selleck Chemicals, S1478), 100 nM bafilomycin A_1_ (Sigma-Aldrich, 196000), cycloheximide (100 μg/ml, MedChemExpress, HY-12320), and MG132 (10 μM, Calbiochem, 474787).

### Mitochondria isolation and proteinase K assay

The cell mitochondria isolation kit (Beyotime Biotechnology, C3601), Dounce tissue grinder (KIMBLE, 885300), and differential centrifugation were utilized to separate mitochondria and cytosolic proteins from whole cell lysates. Briefly, the cells were harvested and lysed, and the cell lysates were then homogenized and centrifuged at 600*g* for 10 min at 4 °C to remove debris. Purified homogenates were centrifuged at 11,000*g* for 10 min at 4 °C. The precipitations were cell mitochondria, and the supernatants were further centrifuged at 12,000*g* for 10 min at 4 °C. The further obtained supernatants were cytosolic proteins. For the proteinase K (BBI, PB0451) treatment, the isolated mitochondria were treated with 10 μg/ml proteinase K for 30 min on ice. Then, the samples were analyzed by SDS-PAGE and immunoblot analysis. The data were representative of three independent experiments.

### Immunoblot and immunoprecipitation analysis

Immunoblot analysis was previously described (30, 31, 32, 33, 34, 35). Briefly, the harvested cells were lysed by the cell lysis buffer, which consisted of 150 mM NaCl, 1% NP-40, 25 mM Tris-HCl (pH 7.6), and 1% sodium deoxycholate containing protease inhibitor cocktail (Roche, 4693132001). After separated by SDS-PAGE, the proteins in cell lysates were transferred onto PVDF membrane (Merck Millipore, IPVH00010) and subjected to immunoblot analysis. For the immunoprecipitation analysis, the lysed cell lysates were centrifuged at 12,000*g* for 15 min at 4 °C, and the supernatants were incubated with Protein G Magnetic Beads (Invitrogen, 10004D) at 4 °C for 4 h which were preincubated with indicated antibodies. Then, the beads were washed three times with cell lysis buffer and further used for immunoblot analysis. The data of immunoprecipitation analysis were representative of three independent experiments. The following antibodies were used: anti-GFP antibody (Santa Cruz Biotechnology, sc-8334), anti-GFP antibody (Santa Cruz Biotechnology, sc-9996), anti-FLAG antibody (Sigma, F3165), anti-GST antibody (Santa Cruz Biotechnology, sc-138), anti-His antibody (Santa Cruz Biotechnology, sc8036), anti-PINK1 antibody (Cell Signaling Technology, 6946), anti-GAPDH antibody (Proteintech, 60004-1-Ig) anti-Tubulin antibody (Proteintech, 11224-1-AP), anti-TOM20 antibody (Proteintech, 11802-1-AP), anti-COXIV antibody (Proteintech, 11242-1-AP), anti-HSP60 antibody (Proteintech, 66041-1-Ig), anti-LC3 antibody (Novus Biologicals, NB1-02200), anti-Ub antibody (Santa Cruz Biotechnology, sc-8017), anti-p-Ub (S65) antibody (Cell Signaling Technology, 37642), anti-PHB2 antibody (Santa Cruz Biotechnology, sc-133094), anti-PHB2 antibody (Proteintech, 12295-1-AP), anti-Parkin (Cell Signaling Technology, 4211), anti-IgG(M), and anti-IgG(R). Secondary antibodies including goat anti-mouse and anti-rabbit IgG (Jackson ImmunoResearch, 111–035–062 and 115–035–045) were used subsequently. Immunoblots were visualized using the ECL detection kit (Thermo, 34,080).

### Mt-mKeima, immunofluorescence analysis, and live cell imaging

Mt-mKeima mitophagic and immunofluorescent analysis were previously described (16). Briefly, HEK293 cells and primary cortical neurons were transfected with mt-mKeima plasmid using lipofectamine 2000 reagent or infected with lentiviral mt-mKeima (Public Protein/Plasmid library, LV01230-2a) for 8 h and were treated with A/O. Then, the neurons were analyzed using confocal microscopy. Immunofluorescent assay was performed similarly to the previous studies (36, 37, 38). In detail, cells were first fixed by 4% paraformaldehyde and permeabilized with 0.1% Triton X-100, and then the cells were subjected to immunofluorescencent staining using primary antibodies and fluorescent secondary antibodies. The following primary antibodies were used: TOM20 antibody (Proteintech, 11802-1-AP) and FLAG antibody (Sigma, F3165). In most cases, the cells were visualized using Zeiss (LSM710) confocal microscope (39, 40, 41), and in some cases using Nikon fluorescent microscope or Nikon (A1R) confocal microscope (42). All of the imaging analyses were carried out by investigator blinded to the identities of the experimental groups.

### In situ PLA

HEK293 cells were fixed with 4% paraformaldehyde and permeabilized with 0.1% Triton X-100. After incubation with primary antibodies, the cells were subjected to the Duolink *in situ* PLA according to the manufacturer’s instructions (Duolink *In Situ* Red, Sigma-Aldrich, duo921001). The following primary antibodies were used: FLAG antibody (Sigma, F3165), PHB2 antibody (Santa Cruz Biotechnology, sc-133094), LC3B antibody (Novus Biologicals, NB1-02200), IgG(M) antibody, and IgG(R) antibody. Images were captured with a Nikon (Ti2) microscope.

### In vitro protein purification and binding assays

The expressed GST-fusion and His-fusion proteins in *E.coli* were separately purified by BeyoGold GST-tag Purification Resin and BeyoGold His-tag Purification Resin (Beyotime Biotechnology, P2251 and P2233) for 1 h at 4 °C. After washed for three times with ice-cold PBS, purified proteins were incubated with second protein for 2 h at 4 °C. After incubation, the beads were washed for four times with 1 × PBS and then were boiled in SDS sample buffer and detected by immunoblot analysis. The data of *in vitro* protein purification and binding assay were representative of three independent experiments.

### Statistical analysis

Immunoblot densitometry of three independent experiments was performed by Photoshop 7.0 (Adobe) software. Cellular localization of PHB2, LC3, and Parkin was determined by visual inspection. Prism 8.0 (GraphPad) software was used to generate charts of the obtained data. *p*-values were displayed in figure legends.

## Data availability

All data described have been included in the manuscript.

## Supporting information

This article contains supporting information.

## Conflict of interest

The authors declare that they have no conflicts of interest with the contents of this article.
