# Supplementary material for: The interaction between E3 ubiquitin ligase Parkin and mitophagy receptor PHB2 links inner mitochondrial membrane ubiquitination to efficient mitophagy
Source: J Biol Chem. 2022 Nov 12;298(12):102704. doi: 10.1016/j.jbc.2022.102704 (PMC9763867; doi:10.1016/j.jbc.2022.102704)
Supplement: Supporting information [file mmc1.pdf]

Supporting Information

The interaction between E3 ubiquitin ligase Parkin and mitophagy receptor PHB2 links inner mitochondrial membrane ubiquitination to efficient mitophagy

Shan Sun, Hongyu Hou, Guoqiang Ma, Qilian Ma, Ningning Li, Li Zhang, Chunsheng Dong, Mian Cao, Kin Yip Tam*, Zheng Ying*, Hongfeng Wang*

Figure S1:

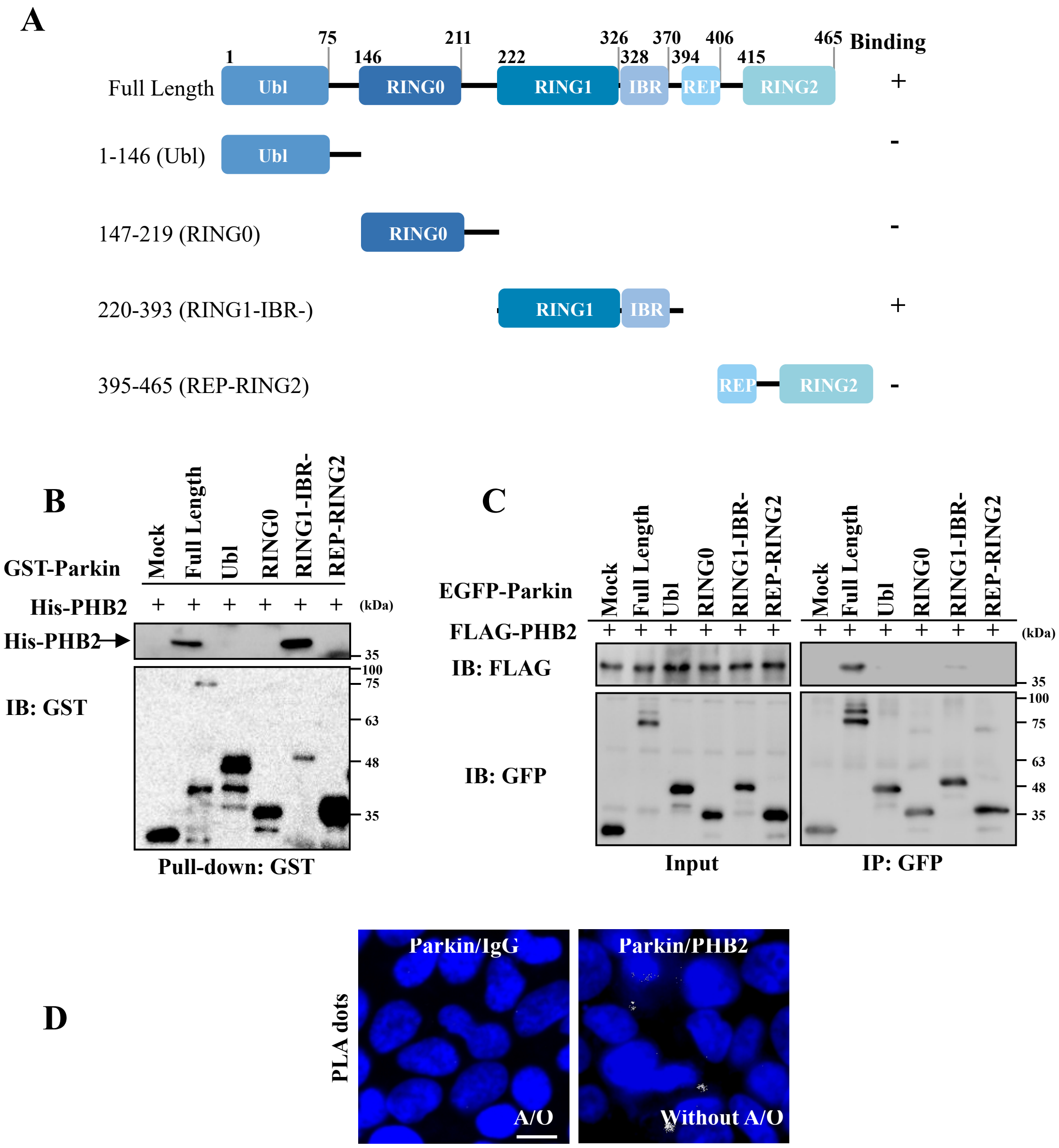

Figure S1. Identification of the domain required for Parkin-PHB2 interaction. (A) Schematic diagram of Parkin constructs used in this study. **(B)** After purification by GST binding resin, different constructs of GST-Parkin (as indicated in A) were separately mixed with His-PHB2. Bound proteins were analyzed by immunoblot analysis with anti-GST and anti-PHB2 antibodies. **(C)** Different constructs of EGFP-Parkin (as indicated in A) and FLAG-PHB2 were transfected into HEK293T cells, and then were treated with 5 μg/ml A/O for 4 h. Cell lysates were processed for immunoprecipitation analysis using anti-GFP antibody and then were analyzed with anti-GFP and anti-FLAG antibodies. **(D)** HEK293 cells expressing EGFP-Parkin were treated with or without 5 μg/ml A/O for 4 h, and then the cells were fixed and stained with anti-GFP and anti-IgG (R) or anti-PHB2 antibody in PLA experiment. The cells were visualized using Nikon microscope. Blue: nuclei (DAPI); white dots: PLA positive puncta. Scale bar, 10 μm.

Figure S2:

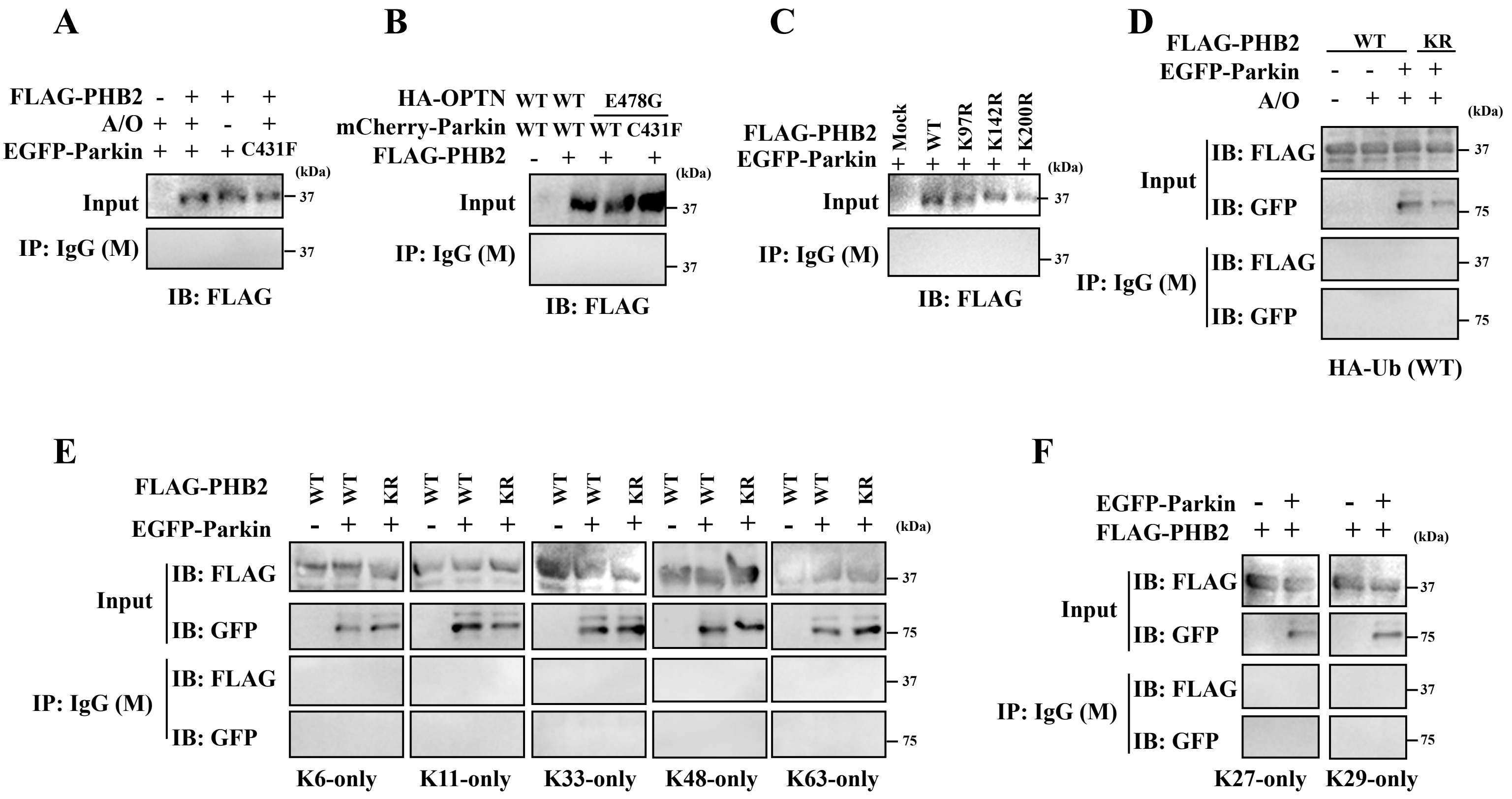

Figure S2. IgG control experiments related to Figure 3. (A) FLAG vector or FLAG-PHB2 were transfected into HEK293T cells with WT or C431F mutant EGFP-Parkin respectively, followed by treatment with or without 5 μg/ml A/O for 4 h. Cell lysates were subjected to immunoprecipitation analysis using anti-IgG (M) antibody, and then were detected using anti-FLAG antibody. **(B)** WT or E478G mutant HA-OPTN, WT or C431F mutant mCherry-Parkin, and FLAG vector or FLAG-PHB2 were transfected into si-OPTN HEK293T cells respectively, followed by treatment with 5 μg/ml A/O for 4 h. Cell lysates were subjected to immunoprecipitation analysis using anti-IgG (M) antibody, and then were detected using anti-FLAG antibody. **(C)** FLAG vector or WT, K97R, K142R, K200R mutant FLAG-PHB2 were transfected into HEK293T cells, along with EGFP-Parkin respectively, followed by treatment with 5 μg/ml A/O for 4 h. Cell lysates were subjected to immunoprecipitation analysis using anti-IgG (M) antibody, and then were detected using anti-FLAG antibody. **(D)** EGFP vector or EGFP-Parkin, and WT or KR mutant FLAG-PHB2 were transfected into HEK293T cells respectively, along with WT HA-Ub, and then the cells were treated with or without 5 μg/ml A/O for 4 h. Cell lysates were subjected to immunoprecipitation analysis using anti-IgG (M) antibody, and then were detected using indicated antibodies. Here and hereafter KR mutant FLAG-PHB2 indicates K142R/K200R mutant FLAG-PHB2. **(E)** EGFP vector or EGFP-Parkin, along with WT or KR FLAG-PHB2 were transfected into HEK293T cells with HA-Ub (K6, K11, K33, K48 or K63-only) respectively, followed by treatment with 5 μg/ml A/O for 4 h. Cell lysates were subjected to immunoprecipitation analysis using anti-IgG (M) antibody, and then were detected using indicated antibodies. **(F)** The similar experiment (without KR mutant FLAG-PHB2) were separately performed in HA-Ub (K27 or K29-only) as **(E)**.

Figure S3:

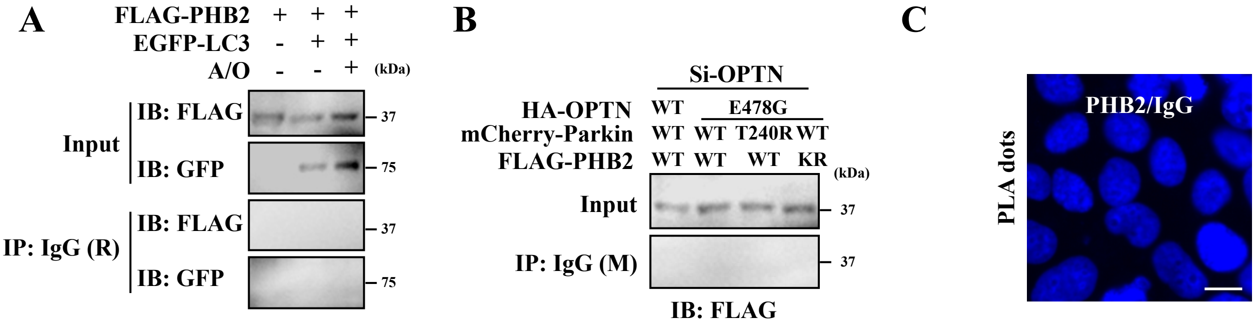

Figure S3. IgG control experiments related to Figure 4. (A) HEK293T cells expressing EGFP vector or EGFP-LC3 with FLAG-PHB2 were treated with DMSO or 5 μg/ml A/O for 4 h. Cell lysates were subjected to immunoprecipitation analysis using anti-IgG (R) antibody, and then were detected using indicated antibodies. **(B)** WT or E478G mutant HA-OPTN, WT or T240R mutant mCherry-Parkin, WT or KR mutant FLAG-PHB2 with EGFP-LC3 were transfected into si-OPTN HEK293T cells, followed by treatment with 5 μg/ml A/O for 4 h. Cell lysates were subjected to immunoprecipitation analysis using anti-IgG (M) antibody, and then were detected using anti-FLAG antibody. **(C)** WT FLAG-PHB2 and HA-Parkin were transfected into HEK293 cells, followed by treatment with 5 μg/ml A/O for 4 h. Then the cells were fixed and stained with anti-FLAG and anti-IgG (R) antibody in PLA experiment. The cells were visualized using Nikon microscope. Blue: nuclei (DAPI); white dots: PLA puncta. Scale bar, 10 μm.

Figure S4:

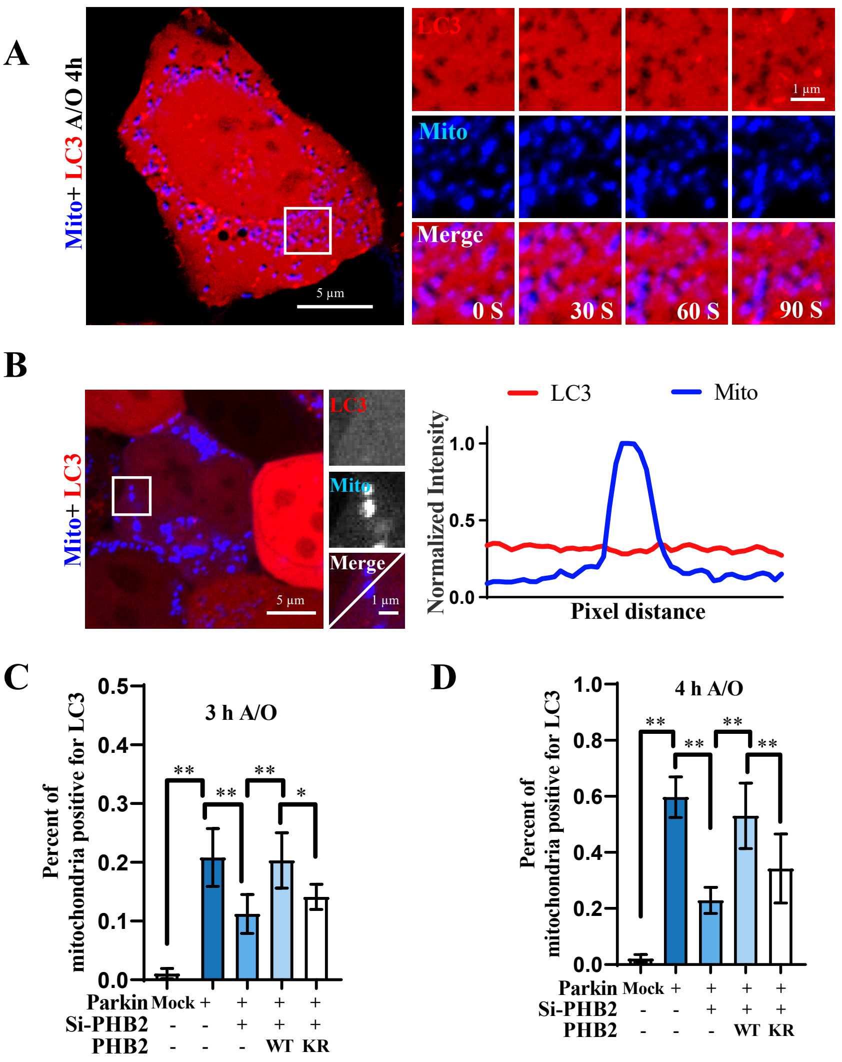

Figure S4. Control experiments and quantification data related to Figure 5. (A-B) HEK293 cells expressing BFP-Mito, EGFP-OPTN and mCherry-LC3 were treated with 5 μg/ml A/O for 6 h. The cells were analyzed by multi-channel live cell imaging using confocal microscopy. **(A)** Regions in white box was magnified on the right side. **(B)** The fluorescence intensity of the paint line (white line) was measured. **(C-D)** FLAG vector or FLAG-Parkin, BFP-Mito, EGFP-OPTN and mCherry-LC3 (with or without WT or KR mutant FLAG-PHB2) were transfected into control or si-PHB2 HEK293 cells. The cells were treated with 5 μg/mL A/O for 3 h (C) or 4 h (D), and then LC3 "rings" co-localization with mitochondria was qualified. Data were collected from three independent experiments and were represented as means ± S.D., **, P<0.01; *, P<0.05, *One-way ANOVA* followed by post hoc Tukey's tests.

Figure S5:

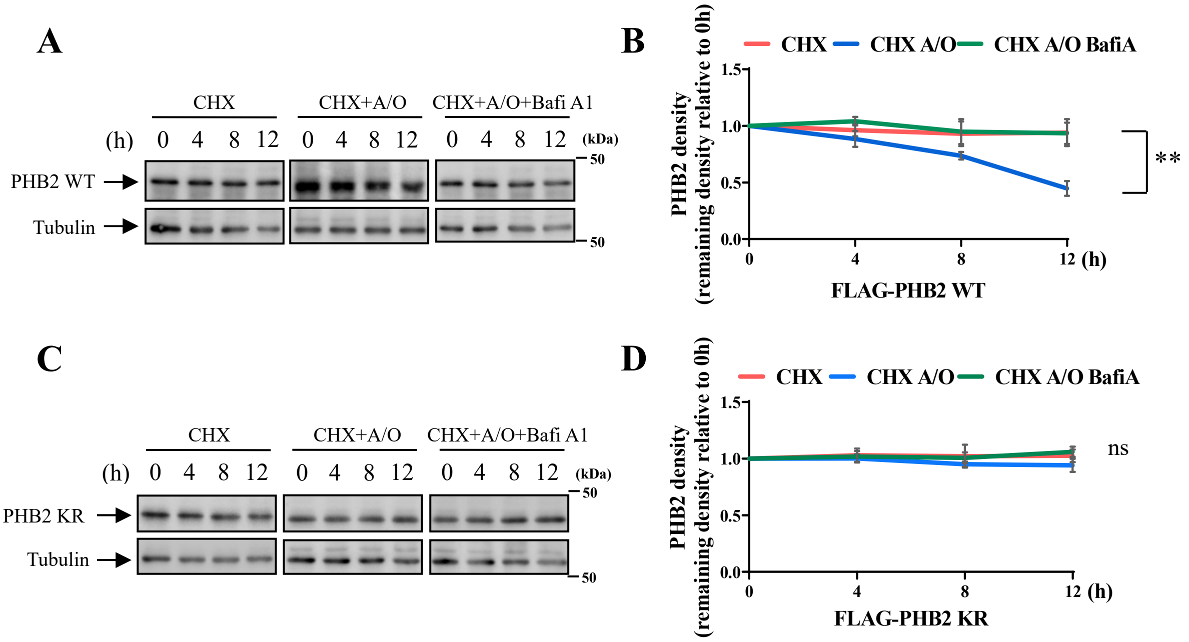

Figure S5. PHB2 is degraded by lysosomal pathway. (A-D) HEK293T cells expressing WT or KR mutant FLAG-PHB2 with mCherry-Parkin were treated with cycloheximide (CHX, 100 µg/ml), along with or without A/O (5 µg/ml) or bafilomycin A_1 (Bafi A1, 100 nM) for 0 h, 4 h, 8 h, 12 h. Then the cell lysates were analyzed by immunoblot analysis with anti-FLAG and anti-Tubulin antibodies. **(B)** and **(D)** separately showed the quantification of protein level about FLAG-PHB2-WT **(A)** and FLAG-PHB2-KR **(C)** at the indicated times. Data from three independent experiments were represented as means ± S.D., **, p<0.01; ns, not significantly, *One-way ANOVA* followed by post hoc Tukey's tests..

Figure S6:

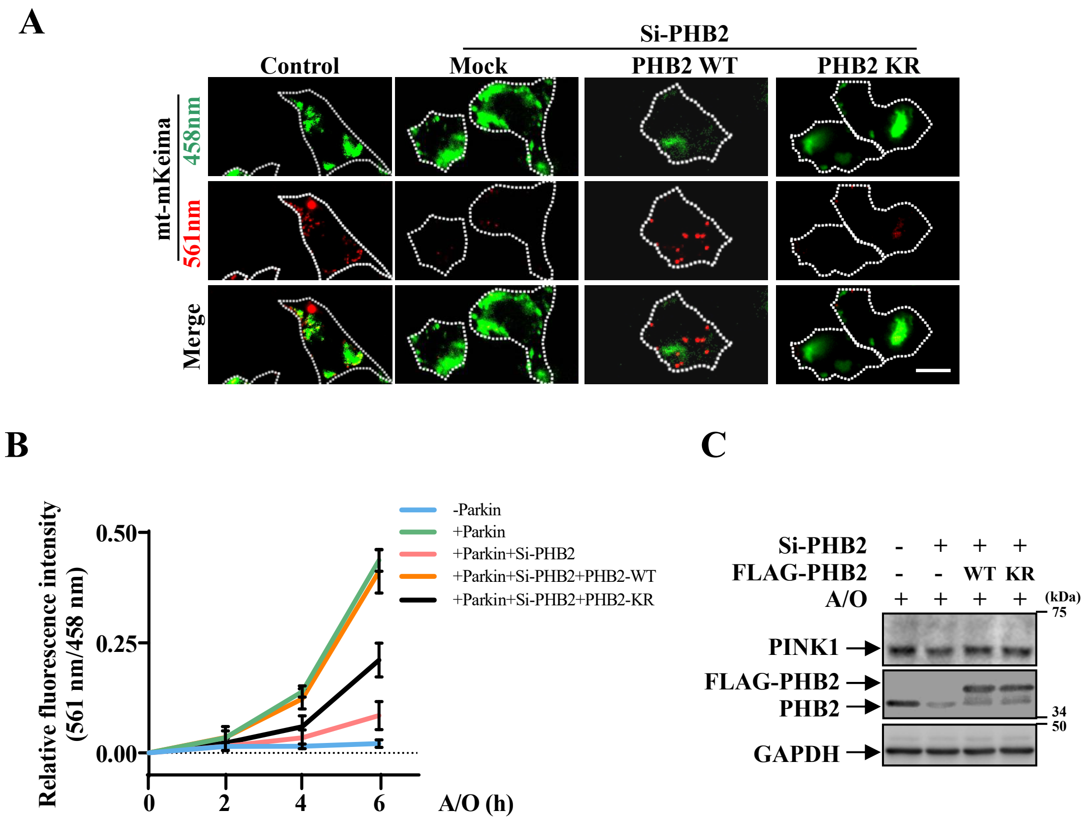

Figure S6. Parkin-mediated ubiquitination of PHB2 is required for efficient mitophagy. (A) SH-SY5Y cells were transfected with control or si-PHB2 and then were re-transfected with WT or KR mutant FLAG-PHB2 with EGFP-Parkin and mt-mKeima. The cells were treated with 5 µg/mL A/O for 4 h, and then were analyzed by confocal microscopy. Scale bars, 10 µm. (**B)** HEK293 cells were transfected with control or si-PHB2 and then were re-transfected with WT or KR mutant FLAG-PHB2 and mt-mKeima with or without EGFP-Parkin. The cells were treatment with 5 µg/ml A/O for 0, 2, 4 or 6 h, and then relative fluorescence intensity (561 nm/458 nm) was quantified. Data from three independent experiments were represented as means ± S.D. **(C)** FLAG vector, WT or KR mutant FLAG-PHB2 were transfected into control or si-PHB2 HEK293T cells, and then the cells were treated with 5 µg/mL A/O for 4 h. Cell lysates were analyzed by immunoblot analysis with anti-PINK1, anti-PHB2 and anti-GAPDH antibodies.
